# Fast functional imaging of multiple brain regions in intact zebrafish larvae using Selective Plane Illumination Microscopy

**DOI:** 10.1186/1471-2202-14-S1-P97

**Published:** 2013-07-08

**Authors:** Raphaël Candelier, Thomas Panier, Sebastián Romano, Raphaël Olive, Thomas Pietri, Germán Sumbre, Georges Debrégeas

**Affiliations:** 1 CNRS / UPMC Univ. Paris 06, FRE 3231, Laboratoire Jean Perrin LJP, F-75005, Paris, France; 2 Ecole Normale Supérieure, Institut de Biologie de l'ENS, IBENS, Paris, F-75005 France; 3 Inserm, U1024, Paris, F-75005 France; 4 CNRS, UMR 8197, Paris, F-75005 France; 5 IBENS, ENS, Paris, France

## 

**Figure 1:**
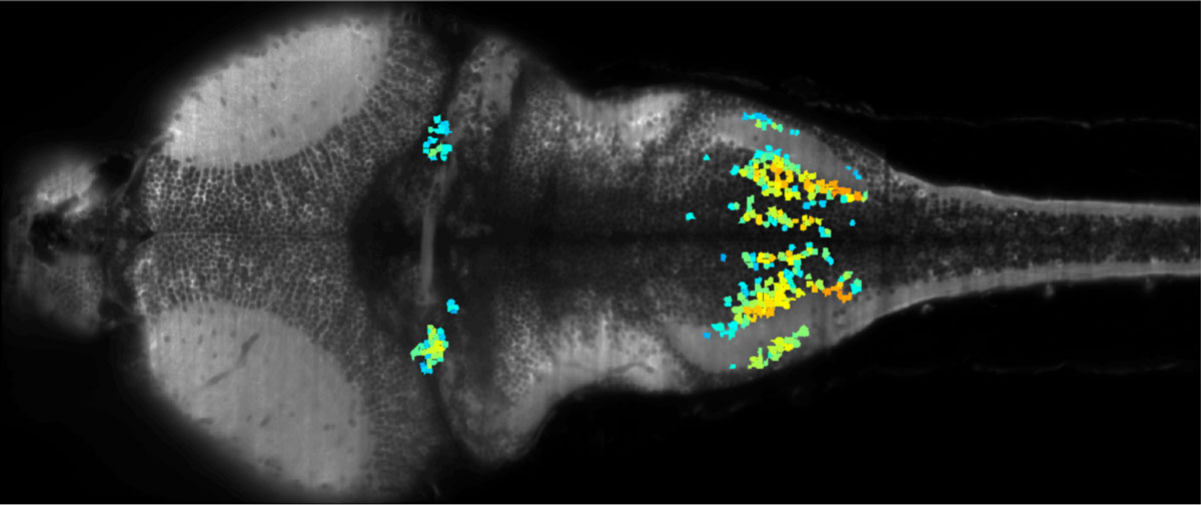
**Image of the brain of a 6 day-old GCaMP3 zebrafish obtained by SPIM**. Colored neurons indicate a set of neurons showing correlated activity.

The optical transparency and the small dimensions of zebrafish at the larval stage make it a vertebrate model of choice for brain-wide *in-vivo *functional imaging. However, current point-scanning imaging techniques, such as two-photon or confocal microscopy, impose a strong limit on acquisition speed which in turn sets the number of neurons that can be simultaneously recorded [1]. At 5 Hz, this number is of the order of one thousand, *i.e*. approximately 1-2% of the brain. We demonstrate that this limitation can be greatly overcome by using Selective-Plane Illumination Microscopy (SPIM) [2-4]. Zebrafish larvae expressing the genetically encoded calcium indicator GCaMP3 were illuminated with a scanned laser sheet and imaged with a camera whose optical axis was oriented orthogonally to the illumination plane. This optical sectioning approach was shown to permit functional imaging of most of the brain volume of 5-9 day old larvae with single-cell resolution. The spontaneous activity of up to 5000 neurons was recorded at 20 Hz for 20-60 min. By rapidly scanning the specimen in the axial direction, the activity of 25000 individual neurons from 5 different z-planes (approximately 30% of the entire brain) could be simultaneously monitored at 4 Hz. Compared to point-scanning techniques, this imaging strategy thus yields a ~20-fold increase in data throughput (number of recorded neurons times acquisition rate) without compromising the signal-to-noise ratio. The extended field of view offered by the SPIM method allowed us to directly identify large scale ensembles of neurons, spanning several brain regions (see Figure 1), that displayed correlated activity and were thus likely to participate in common neural processes.
